# *Ca^2^Lib*: Simple and Accurate LiDAR-RGB Calibration Using Small Common Markers

**DOI:** 10.3390/s24030956

**Published:** 2024-02-01

**Authors:** Emanuele Giacomini, Leonardo Brizi, Luca Di Giammarino, Omar Salem, Patrizio Perugini, Giorgio Grisetti

**Affiliations:** Department of Computer, Control, and Management Engineering “Antonio Ruberti”, Sapienza University of Rome, 00185 Rome, Italy; brizi@diag.uniroma1.it (L.B.); digiammarino@diag.uniroma1.it (L.D.G.); salem@diag.uniroma1.it (O.S.); perugini.1844358@studenti.uniroma1.it (P.P.)

**Keywords:** LiDAR, camera, extrinsic calibration

## Abstract

Modern visual perception techniques often rely on multiple heterogeneous sensors to achieve accurate and robust estimates. Knowledge of their relative positions is a mandatory prerequisite to accomplish sensor fusion. Typically, this result is obtained through a calibration procedure that correlates the sensors’ measurements. In this context, we focus on LiDAR and RGB sensors that exhibit complementary capabilities. Given the sparsity of LiDAR measurements, current state-of-the-art calibration techniques often rely on complex or large calibration targets to resolve the relative pose estimation. As such, the geometric properties of the targets may hinder the calibration procedure in those cases where an ad hoc environment cannot be guaranteed. This paper addresses the problem of LiDAR-RGB calibration using common calibration patterns (i.e., A3 chessboard) with minimal human intervention. Our approach exploits the flatness of the target to find associations between the sensors’ measurements, leading to robust features and retrieval of the solution through nonlinear optimization. The results of quantitative and comparative experiments with other state-of-the-art approaches show that our simple schema performs on par or better than existing methods that rely on complex calibration targets.

## 1. Introduction

Integrating Light Detection And Ranging (LiDAR) with RGB imaging systems significantly boosts the fields of vision and perception. The complementary nature of these two vision sensors closes the gap between spatial and visual understanding of the operating environment: LiDAR technology is renowned for its high-accuracy depth-sensing capabilities, providing a foundation for understanding the spatial aspects of the environment. Concurrently, RGB cameras provides high-resolution color information that, on the other hand, allows intelligent systems to understand the visual aspect of the environment. Current research shows that the LiDAR-RGB combination may be fused together using guided depth completionapproaches to provide a unified dense RGB-D representation, which is widely employed for perception applications [1,2,3,4]. Furthermore, in the field of 3D reconstruction, recent findings show that coupling the two sensors may lead to a more robust and accurate estimate [5].

**Figure 1:**
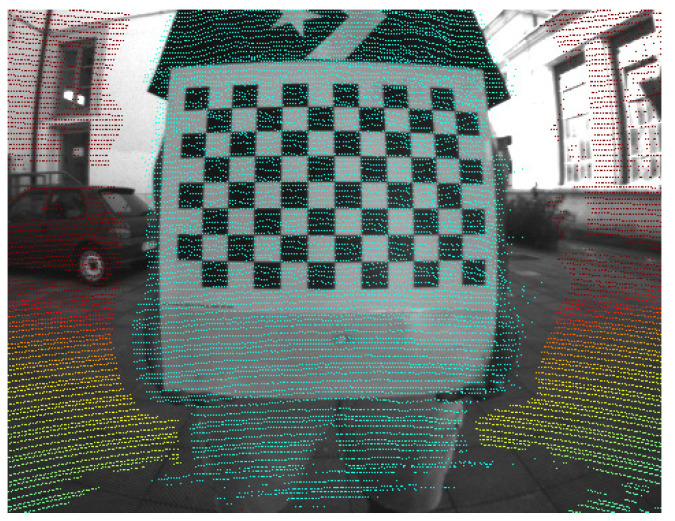
Reprojection of a LiDAR point cloud on a fisheye RGB camera rigidly attached to the former. The offset between the sensors leads to shadows in parts of the image.

Still, integrating the two systems poses a significant challenge due to their inherently different natures. This challenge stems from the need to align and synchronize data streams with different modalities. We focus on the former, estimating the relative positions and orientations between these sensors (extrinsic calibration) using cues extracted from their measurements as shown in Figure 1. Currently, LiDAR-RGB calibration can be solved using a calibration target or marker, as done already for uni-modal sensor extrinsic estimation (i.e., multi-RGB [6]). The calibration target represents a unique object whose geometric and visual properties are known and, through tailored detection algorithms, can be measured precisely. Identifying common elements from both viewpoints is typically sufficient for determining spatial correlations between sensors. However, discerning which elements are relevant in this scenario remains problematic. Due to the sensors’ varying resolutions and visual patterns, traditional markers such as checkerboard corners are not viable. This necessitates the exploration of more complex features. One example could be the use of holes of known dimensions in the target: LiDARs would be able to infer the center of the hole by measuring the points on the border, while cameras could estimate the same point using typical circle detection algorithms [7]. Although these features have proven effective for extrinsic estimation, the requirement of ad hoc calibration targets poses more practical and often economical problems (these specific markers are often realized using CNC printers). Moreover, calibration is often carried out directly onsite, so the target’s portability and size are other problems that must be discussed. This paper introduces a method for extracting robust planar features readily obtainable from sensor data. We relax the requirements for calibration targets to typical patterns already used for RGB calibrations (i.e., checkerboards or ChAruCO [8]) with sizes down to A3/A4 dimensions for portability. Leveraging a direct nonlinear formulation, we can achieve a highly accurate relative pose estimate even with a minimum of three observations per sensor. Finally, we release an open-source implementation of our toolbox.

The paper is structured as follows: Section 2 provides a comprehensive literature review highlighting previous works in LiDAR-RGB calibration. In Section 3, we describe preliminary concepts required to understand our approach, followed by details of our calibration pipeline. Section 4 describes the conducted experiments, their setup, data collection, and results. Finally, Section 5 summarizes our key contributions and potential limitations and suggests directions for future research.

## 2. Related Work

In this section, we delve into the field of LiDAR-RGB calibration and explore the two principal methodologies: *target-based* and *target-less* approaches. As the name suggests, target-based approaches require the user to place artificial markers that both the camera and LiDAR can easily detect. This contrasts with target-less methods that free the user from this task. The core idea of calibration is common in the two approaches: computing common features between heterogeneous measurements and estimating the transformation that minimizes the distances between corresponding features.

First, an overview of target-less approaches is presented: Pandey et al. present an automatic data-driven approach based upon the maximization of mutual information between the sensor-measured surface intensities [9]. Using different calibration parameters, the authors exploit the correlation coefficient for many scan–image pairs’ reflectivity and intensity values. However, shadows of objects or colored surfaces that completely absorb infrared light might result in a weaker correlation between scan–image pairs. Yoon et al. propose a calibration method using region-based object pose estimation. Objects are segmented in both measurements, and then a 3D mesh is generated from the LiDAR measurements, while images are used to reconstruct the scene using Structure from Motion (SfM). The two models are then registered together to acquire an initial guess of the relative pose. The final solution is obtained iteratively by finding correspondences between the reconstructed objects from both measurements [10]. Bai et al. introduce an approach for calibration that relies on parallel lines commonly visible in urban environments (i.e., edges of buildings) [11]. The relative pose estimate is obtained by aligning the directions of the lines observed by the two sensors to find the relative orientation and then by solving a set of linear point-on-line constraints to find the relative translation. In recent years, the development of learning-based methods has also expanded into this field: Lv et al. propose a real-time self-calibration network that predicts the extrinsic parameters by constructing a cost volume between RGB and LiDAR features [12], while Sun et al. first estimate an initial guess by solving a hand–eye calibration method [13]. Moreover, the guess is fine-tuned by segmenting the image–cloud pair and aligning the distances between centroids. The advantage of the target-less method is that it can be used without preparing the environment. This comes at the cost of lower accuracy and robustness when compared to their target-based counterparts. Target-based methods estimate the relative pose using an observed known structure. Given the difference in resolution for the two sensors, it is highly unlikely that correspondences within the measurements can be established directly. For this reason, point-to-point methods tend to process LiDAR measurements to implicitly obtain virtual points (points that are not explicitly detected but are estimated from the LiDAR measurement) easily detectable from an RGB sensor. For instance, Park et al. utilize a specially designed polygonal planar calibration board with known lengths of adjacent sides [14]. By estimating the 3D corresponding points from the LiDAR, vertices of the board can be determined as the meeting points of two projected sides. The vertices, along with the corresponding points detected from the color image, are used for calibration. Pusztai et al. introduce a methodology that utilizes cubic objects with predetermined side lengths [15,16]. The corners of the cubes are estimated by initially detecting each side of the box and subsequently determining their intersection points. Furthermore, the corners and their corresponding RGB image are employed to calibrate the system by solving Iterative Corresponding Point (ICP). Zhou et al. propose a single-shot calibration method requiring a checkerboard [17]. The target is detected both in the RGB image and LiDAR measurement, using RANSAC [18] for the latter. Furthermore, the four edges of the checkerboard are estimated and aligned to compute the relative offset between the two sensors. Grammatikopoulos et al. use a custom-made retro-reflective target paired with an AprilTag [19] fiducial marker to establish correspondence at the center of the target [20]. The relative pose is optimized by solving a Perspective-n-Points (PnP) problem. Tóth et al. introduce a fully automatic calibration technique that leverages the utilization of spheres: enabling accurate detection in both point clouds and camera images [21]. Upon successful detection, the algorithm aligns the set of sphere centers using SVD. Beltrán et al. present a methodology that utilizes a custom calibration target with four holes and AruCO markers specifically designed for monocular detection [22]. The methodology employs a set of techniques for each sensor to estimate the center points of the holes. Subsequently, the relative offset between sensors is determined by aligning the set of centers obtained from each sensor. Li et al. adopt a similar approach by using a checkerboard with four holes [23]. Fan et al. propose a two-stage calibration method using an auxiliary device with distinctive geometric features [24]. The method extracts lines from images and LiDAR point clouds to provide an initial estimation of the external parameters. Nonlinear optimization is then applied to refine these parameters. In the work of Singandhupe et al., the authors first extract planar information from RGB and LiDAR measurements; then, two grids of points are extracted from the computed planar patches and aligned using a customized ICP algorithm [25]. Albeit these approaches provide accurate results with few measurements, care should be taken during the estimation of virtual correspondences, as they can cause significant errors in the estimation step. Moreover, these custom targets often require precise construction or expensive manufacturing.

Another group of approaches does not directly solve the calibration problem using point-to-point correspondences but rather exploits the flatness of the target to reduce the feasible set of solutions using plane-to-plane constraints. Mirzaei et al. address the challenge of acquiring accurate initial estimates by dividing the problem into two sub-problems and analytically solving each to obtain precise initial estimates [26]. The authors then refine these estimates through iterative minimization. They also discuss the identifiability conditions for accurate parameter estimation. Finally, in a method similar to our proposal, Kim et al. combine observed normals first to estimate the relative orientation with SVD and then iteratively estimate an initial guess of the relative translation by minimizing the pairwise planar distances between measurements [27]. Finally, the translation is refined using a nonlinear optimization problem using Levenberg-Marquardt (LM). Despite its simplicity, this method decouples the estimation of orientation and translation, thus leading to potential losses in accuracy while also increasing the minimum number of measurements for an acceptable solution.

Compared with the state-of-the-art, we propose:

- A formulation for joint nonlinear optimization that couples relative rotation and translation using a plane-to-plane metric;
- An extensible framework that decouples optimization from target detection and currently supports checkerboard and ChARuCO patterns of typical A3–A4 sizes, which are easily obtainable from commercial printers;
- The possibility to handle different camera models and distortion;
- An open-source implementation.

## 3. Our Approach

This section provides a detailed and comprehensive description of our method. First, we describe the preliminaries required to understand our approach, and then every pipeline component is described, following the procedure from the acquisition of the measurements up to the computation of the relative poses between the two sensors (extrinsic parameter).

**Figure 2:**
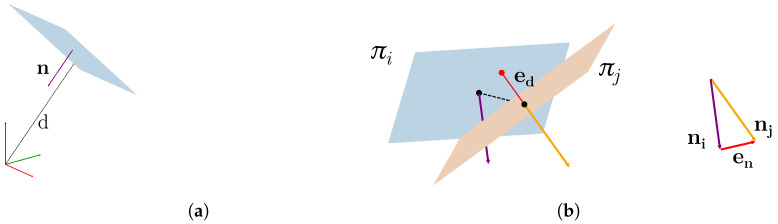
Here, $e_{n}$ is the error for the normal term while $e_{d}$ represents the plane distances. (**a**) shows the plane representation used in this work. (**b**) A visual representation of the plane-to-plane error.

**Plane Representation:** Let π=(n,d) be a 3D plane, where $n\in S^{2}$ represents its normal and d∈R is its orthogonal distance concerning the origin coordinate system (visible in Figure 2a). Applying a transform X∈SE(3) to a plane π yields new coefficients $\pi^{\prime }$ as follows:

(1) $$X\pi =\left\{\begin{array}{c} Rn\\ d+{\left(Rn\right)}^{T}t \end{array}\right.$$

Here $X=\langle R;t\rangle$ is represented by a rotation matrix R∈SO(3), and the translation vector $t\in R^{3}$.

We represent as Δx the Lie algebra se(3) associated with the group SE(3), parameterized as $\Delta x={\left[\Delta t,\Delta r\right]}^{T}$; $\Delta t\in R^{3}$ is the translation, and $\Delta r\in R^{3}$ is the rotation expressed in angle–axis representation. The rotation matrix can be calculated from the perturbation vector using the exponential map at the identity exp(Δr)∈SO(3). We extend the notation of the exponential map to refer to the transformation encoded in Δx. If the transformation is modified by a small local perturbation Δx=(Δr|Δt), then we can rewrite:

(2) $$\left(X\;\exp\left(\Delta x\right)\right)\pi =\left\{\begin{array}{c} \exp(\Delta r)Rn\\ d+{\left(Rn\right)}^{T}t+n^{T}R^{T}\exp{\left(\Delta r\right)}^{T}\Delta t \end{array}\right.$$

Deriving the result for Δx leads to the following Jacobian:

(3) $$\frac{\partial (X\;\exp(\Delta x))\pi }{\partial \Delta x}={\left[\begin{array}{ccc} \; & 0_{3\times 3} & -{\left\lfloor Rn\right\rfloor }_{\times }\\ \; & n^{T}R^{T} & 0_{1\times 3} \end{array}\right]}_{4\times 6}$$

where ${\left\lfloor v\right\rfloor }_{\times }$ maps the vector v into a skew-symmetric matrix defined as follows:

(4) $${\left\lfloor v\right\rfloor }_{\times }=\left[\begin{array}{ccc} 0 & -v_{z} & v_{y}\\ v_{z} & 0 & v_{x}\\ -v_{y} & v_{x} & 0 \end{array}\right]$$

The distance between two planes depends on the difference between their normals and the signed distance of the planes from the origin, as shown in Figure 2b. These quantities can be captured by a 4D error vector $e_{p}$ expressing the *plane-to-plane* error metric:

(5) $$\begin{array}{cc} p(\pi_{k}) & =-n_{k}d_{k} \end{array}$$

(6) $$\begin{array}{cc} e_{p}\left(\pi_{i},\pi_{j}\right) & =\left[\begin{array}{c} e_{n}\\ e_{d} \end{array}\right]=\left[\begin{array}{c} n_{i}-n_{j}\\ n_{j}^{T}\left(p\left(\pi_{j}\right)-p\left(\pi_{i}\right)\right) \end{array}\right]. \end{array}$$

Here, $p(\pi_{k})$ is the point on the plane closest to the origin of the reference system, and it is obtained by taking a point along the normal direction n at a distance *d*.

**Pinhole Model (RGB):** Let p be a point expressed in a camera frame and K be the camera matrix. Assuming any lens distortion effect has been previously corrected, then the projection on the image plane of p is computed as

(7) $$\begin{array}{cc} \pi_{c}\left(p\right) & =\phi (Kp) \end{array}$$

(8) $$\begin{array}{cc} K & =\left[\begin{array}{ccc} f_{x} & 0 & c_{x}\\ 0 & f_{y} & c_{y}\\ 0 & 0 & 1 \end{array}\right] \end{array}$$

(9) $$\begin{array}{cc} \phi (v) & =\frac{1}{v_{z}}\left[\begin{array}{c} v_{x}\\ v_{y} \end{array}\right] \end{array}$$

where ϕ(v) represents the homogeneous division and $\pi_{c}\left(p\right)$ the pinhole projection function. For simplicity, we detail only the pinhole camera projection; however, the same principle applies to more complex camera models.

**Figure 3:**
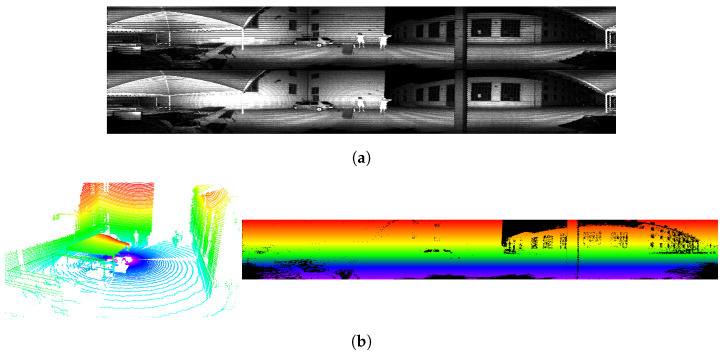
(**a**) Comparison between the spherical projection (top) used for LiDAR images and the projection by ID (bottom) used for this work on intensity information. (**b**) Ring information before (left) and after (right) the projection. Points with the same color have been measured by the same vertical beam throughout the acquisition.

**Projection by ID (LiDAR):** Let p be a point detected by the LiDAR and expressed in its frame. Its projection is computed as:

(10) $$\begin{array}{cc} \pi_{l}\left(p\right) & =A\psi (p) \end{array}$$

(11) $$\begin{array}{cc} A & =\left[\begin{array}{ccc} f_{x} & 0 & c_{x}\\ 0 & 1 & 0 \end{array}\right] \end{array}$$

(12) $$\begin{array}{cc} \psi (v) & =\left[\begin{array}{c} atan2(v_{y},v_{x})\\ \mathrm{ring}(v)\\ 1 \end{array}\right] \end{array}$$

where $f_{x}$ represent the azimuth resolution of the LiDAR, while $c_{x}$ denotes the offset in pixels. The ring(v) function described in Figure 3b is usually provided by the LiDAR and represents the index of the vertical receiver that measured the point. If this information is unavailable and the cloud is ordered, then it is obtainable by dividing the point index by the horizontal resolution of the sensor. Figure 3a shows a comparison with the classical spherical projection. The projection by ID does not preserve the geometric consistency of the scene but provides an image with no holes, which is preferred for computer-vision applications.

**Figure 4:**
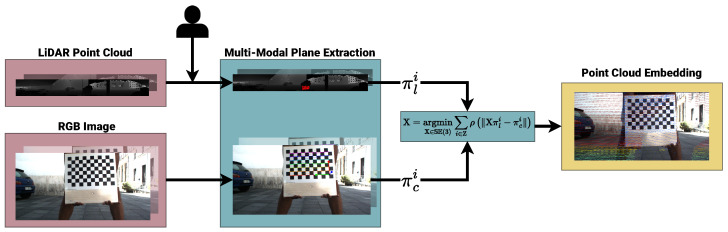
Diagram of our calibration pipeline. Measurements are acquired, and calibration target detection is performed (LiDAR planar detection is performed via human intervention). The set of planes is used to solve the nonlinear optimization problem, leading to the optimal relative pose between the sensors.

As shown in Figure 4, we process the incoming raw LiDAR and RGB measurements to acquire planar information. Assuming the scene remains static throughout the acquisition of a single joint measurement (in this context, a measurement represents a synchronized pair of LiDAR-RGB measurements), the LiDAR cloud is embedded in an image using the projection by ID. Moreover, the system awaits user interaction to guess the position of the calibration target on the LiDAR image.

A parametric circular patch around the user’s selection is used to estimate a plane using RANSAC, and concurrently, the calibration target detection is performed on the RGB image using a target-dependent method (i.e., OpenCV [6]). Once the target is detected, the RGB plane is computed by solving a PnP problem with OpenCV. If the user is satisfied with both the LiDAR and RGB planes, they are stored for processing.

Whereas a straightforward rank analysis of the Jacobians reveals that just three measurements are sufficient to constrain a solution, it is well known from the estimation theory that the accuracy grows with the number of measurements.

Once the set of measurements is acquired, we jointly estimate the relative orientation and translation of the LiDAR to the RGB sensor X∈SE(3) by solving the following nonlinear minimization problem:

(13) $$X=\underset{X\in \mathrm{SE}(3)}{\mathrm{argmin}}\sum_{i\in Z}{∥\underset{e_{p}}{\underset{︸}{X\pi_{l}^{i}-\pi_{c}^{i}}}∥}^{2}$$

where $e_{p}$ represents the plane-to-plane error.

During acquisition, the user may accept one or more wrongly estimated measurements. Due to the quadratic nature of the error terms, these *outliers* are often over-accounted for, resulting in wrong estimations. We employ a Huber *M-estimator* ρ(·) that treats different measurements based on their error to account for this factor. We rewrite Equation (13) as follows:

(14) $$X=\underset{X\in \mathrm{SE}(3)}{\mathrm{argmin}}\sum_{i\in Z}\rho \left(∥X\pi_{l}^{i}-\pi_{c}^{i}∥\right).$$

To resolve Equation (14), we employ the Gauss-Newton (GN) algorithm.

## 4. Experimental Evaluation

In this section, we describe the experiments we conducted to establish the quality of our calibration toolbox. We perform quantitative experiments in the simulated environment provided by [22] to compare our estimates with the ground truth, while we also conduct qualitative and quantitative experiments on real scenarios using our acquisition system. We directly compare our results with [27], as it is the work closest to ours. In addition, we compare to [22], in which the authors produced accurate results by relying on a very complex target (CNC printed).

### 4.1. Synthetic Case

We conduct experiments on the *Gazebo* simulator [28] to evaluate the accuracy and robustness of our approach; we inject different noise figures into the sensor measurements. We also experiment with how the number of observations affects the final results. The scene setup includes a Velodyne HDL-64 LiDAR [29], a BlackFly-S RGB sensor [30], and a 6×8 checkerboard target with a corner size of 0.2 m. We randomly generate and acquire 53 valid measurements (a valid measurement is one for which both the LiDAR and RGB sensor can detect the target).

To quantify the impact of the number of measurements on the accuracy of our approach, we run the calibration procedure with an increasing number of measurements $w_{s}=\left[3\ldots 39\right]$ and at three different LiDAR noise levels $\sigma_{l}$ (0 mm, 7 mm, and 14 mm). For every $w_{s}$, we sample 40 sets of measurements.

From Table 1, we observe a steady decrease in error for every noise level: reaching an average of 2.6 mm translation error in the intermediate noise case. In the case of three measurements, the high uncertainty is due to the potentially poorly conditioned system when using planes with similar normals. Nonetheless, we compare our best result with three measurements against the best results of the methods presented in [22,27]. Table 2 shows the results.

### 4.2. Real Case

**Figure 5:**
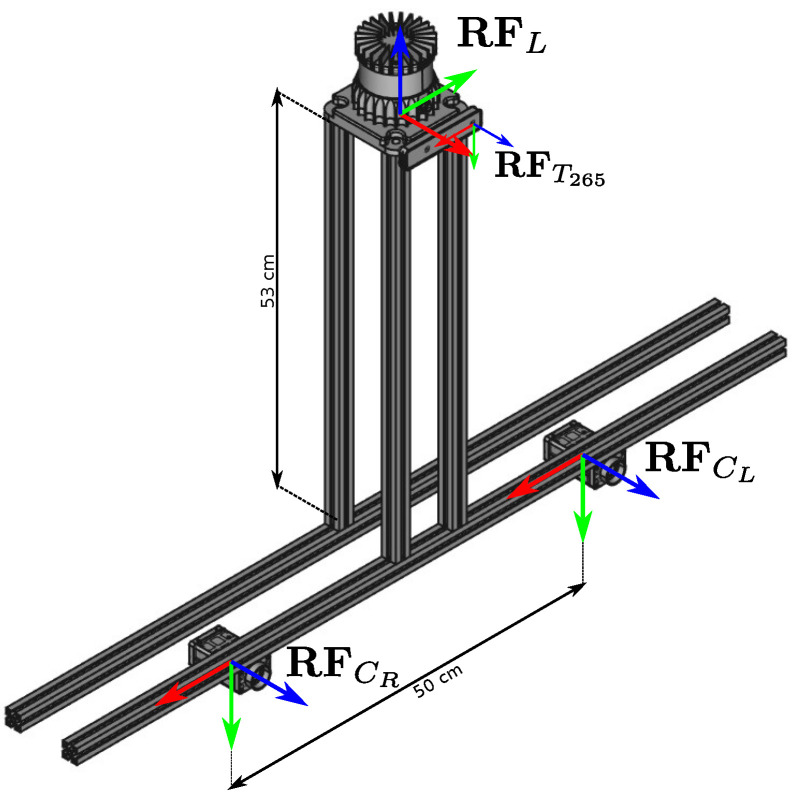
Acquisition system used for the real-case experiments featuring delineated reference systems for each sensor. We report nominal measurements between the sensors. The Realsense T265 ($180^{\circ }$ horizontal field of view) is installed closer to the LiDAR, while the two Manta cameras (90° horizontal field of view) are mounted on a wider horizontal stereo baseline.

In this section, we describe the experiments conducted using real measurements. We perform a quantitative test on our acquisition system shown in Figure 5 that is equipped with an Ouster OS0-128 LiDAR [31] with a resolution of 128×1024, a RealSense T-265 stereo camera, and two Manta-G145 [32] RGB cameras arranged in a wide horizontal stereo configuration.

Since no ground truth information is available, we use the stereo extrinsic to estimate the calibration error. The offset between multiple cameras is measured using optical calibration procedures, which typically reach subpixel precision.

**Figure 6:**
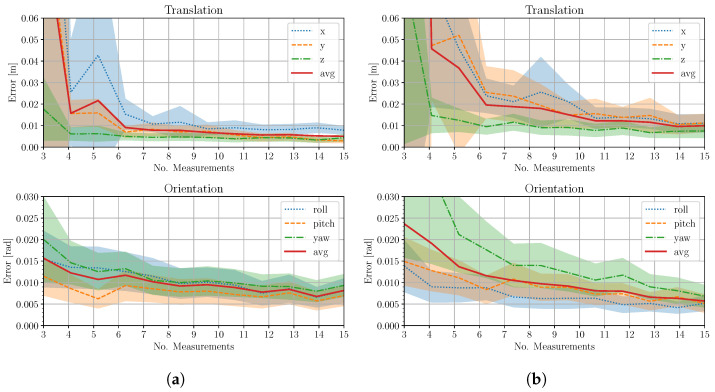
(**a**) Average camera-wise calibration error for the LiDAR-T265, wide fov case. (**b**) Average camera-wise calibration error (standard deviation in shaded color) for the LiDAR-Manta case.

In the first experiment, we consider the LiDAR and the Realsense T-265 sensor [33], which provides two wide field-of-view fish-eye cameras with factory-calibrated intrinsic/extrinsic parameters. The task of the experiment is to demonstrate the accuracy of the calibrator in real-case scenarios and to understand how the number of measurements considered affects the quality of the solution. As in the synthetic case, we first acquire a set of 17 cloud-image LiDAR-RGB measurements for both cameras. Moreover, we perform 40 calibrations with $w_{s}$ randomly selected measurements with $w_{s}\in \left\{3,15\right\}$. Finally, for every $w_{s}$, we combine the computed extrinsics for each camera to obtain an estimated stereo transform. Assuming approximately symmetrical errors in the two cameras, Figure 6a shows the results of this experiment. We obtained, at best, an average error of 7.1 mm in translation and 0.01 rads in orientation.

The second experiment is conducted using the wide stereo setup, for which we also calibrate the intrinsics and extrinsics of the cameras in order to obtain the results expected from a typical scenario. The large parallax between the LiDAR and each camera and the smaller field of view allow us to evaluate our approach in a stressful scenario. The acquisition procedure is the same as in the first experiment, and Figure 6b shows the experimental result, where we obtain the best solution with errors of 4.6 mm in translation and $0.2\times 10^{-2}$ rads in orientation.

**Figure 7:**
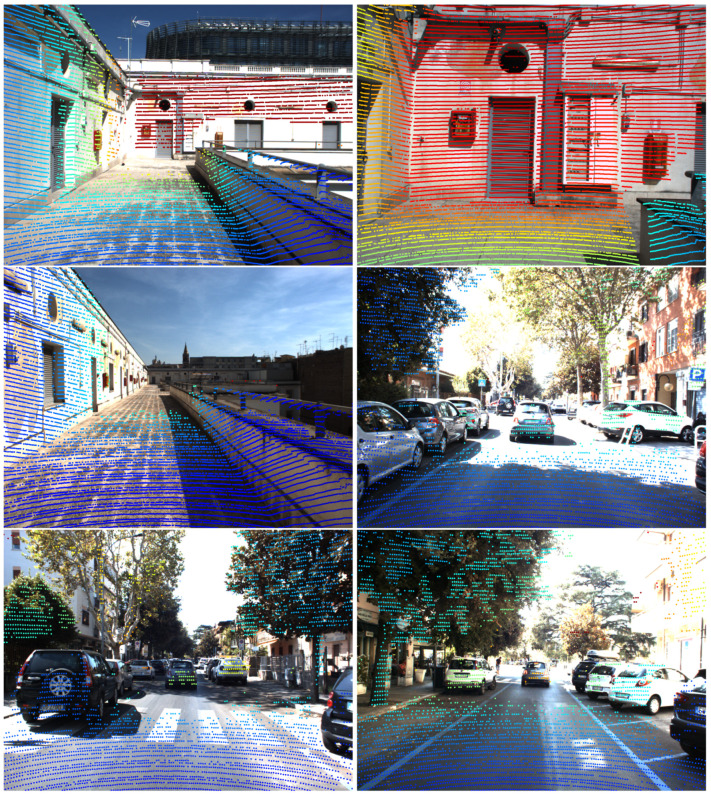
Qualitative samples showing LiDAR cloud projection on RGB image in the setting sketched in Figure 5, for which the parallax between the sensors is approximately 66 cm.

Moreover, Figure 1 and Figure 7, respectively, show the reprojection onto the right camera of the fisheye and wide baseline RGB sensor. For the latter, the large parallax between the sensors leads to strong occlusion effects that have been mitigated with a hidden point removal algorithm [34].

Our evaluation indicates that our method can generate extrinsic estimates comparable or superior to those obtained using other state-of-the-art approaches. It is important to note that careful consideration is required when selecting the minimal number of measurements. However, our experiments demonstrate that the accuracy of these estimates improves as the number of measurements increases.

## 5. Discussion

The experiments show that planar features are a valid alternative to existing solutions for LiDAR-RGB calibrations due to resiliency to LiDAR inherent noise. In particular, Table 1 shows that similar translation error occurs across different noise levels of the sensors. Moreover, real-case experiments support our claim concerning the dimensions of the calibration target, which was brought down to A3/A4 dimensions, along with the seamless integration of different camera models (Kannala-Brandt [35] for T-265 and Rad-Tan for Manta G-145). We suggest using our methodology in situations for which calibration should be performed onsite, where an ad hoc environment for calibration is not guaranteed, or where bringing more-specialized calibration targets is not feasible. An important note regards the sensors’ configuration and shared field of view. Ensuring a correct result requires multiple views of the calibration target from both perspectives. In those cases where the shared field of view is small, a single-shot calibration approach might produce better results in terms of accuracy. Finally, concerning situations where the calibration target is not static during acquisition, caution should be taken for temporal synchronization of the measurements. Our system assumes input measurements to be synchronized; moreover, even small offsets worsen the calibration accuracy. We remark on the difficulty of synchronizing these two sensors due to their different natures. In particular, the revolution period for typical LiDARs is higher compared to the exposure time of RGB sensors. We suggest acquiring RGB images when the LiDAR scan overlaps the camera field of view.

One possible addition that would benefit this work is an automatic detection system for calibration targets in LiDAR measurements. This problem may be tackled from a spatial perspective on the raw point cloud or visually by projecting the cloud onto a 2D embedding. This feature would either fully or partially replace the current human-aided LiDAR plane detection by providing a good initial guess regarding the calibration target’s position.

In conclusion, the paper introduces a simple and effective method for accurately estimating extrinsic parameters between LiDARs and RGB sensors. By leveraging the inherent planar shape of standard calibration patterns, we establish common observations between these sensors to greatly simplify the calibration procedure. Our experiments show that planar features mitigate the LiDAR noise, leading to accurate results even with common A3/A4 calibration patterns. Finally, we also release an open-source implementation to benefit the community.

## Figures and Tables

**Table 1:** Average translation error in millimeters with different noise levels and numbers of measurements *N*.

	$\sigma_{l}=0$ $\sigma_{c}=0$		$\sigma_{l}=8\times 10^{-3}$ $\sigma_{c}=7\times 10^{-3}$		$\sigma_{l}=16\times 10^{-3}$ $\sigma_{c}=14\times 10^{-3}$	
*N*	Mean	Stdev	Mean	Stdev	Mean	Stdev
3	41.761	104.362	20.790	25.124	57.849	112.365
4	10.872	17.941	12.206	12.363	14.940	11.681
5	6.492	7.997	8.350	9.076	9.115	5.675
10	4.591	3.458	5.759	4.974	5.849	1.989
20	2.575	1.981	3.646	2.564	4.123	1.139
30	2.673	1.263	2.867	1.659	3.735	0.878
39	2.091	0.883	2.666	1.206	3.261	0.413

**Table 2:** Quantitative results on synthetic data achieved through calibration using N=3 measurements. The best results are indicated in bold. We choose this measurement count for parity with the methodology proposed in [22]. In [22], a single measurement is deemed sufficient for calibration determination, with 3 measurements considered the optimal scenario. Beyond 3 measurements, accuracy does not improve significantly. Both for our study and in alignment with the findings in [27], 3 measurements represents the minimum requirement for solution determination, and an increase in this count is expected to result in more precise outcomes. Our results show that with our minimum number of measurements, we perform on par with [22] on rotation while outperforming all methods on translation using small commercial tags.

Method	$e_{t}$ (cm)	$e_{r}$ ($10^{-2}$ rad)
Beltrán et al. [22]	0.82	**0.24**
Kim et al. [27]	10.2	129.56
Ours	**0.11**	0.25

## Data Availability

The open-source implementation can be found at https://github.com/rvp-group/ca2lib (accessed on 29 January 2024).
